# Immunomodulatory Effects of Acupuncture on Inflammatory Markers in Patients with Musculoskeletal Pain: A Systematic Review of Randomized Controlled Trials

**DOI:** 10.3390/muscles5020036

**Published:** 2026-05-08

**Authors:** Chi Ngai Lo, Marcus Kwong Lam Fung, Bernard Pui Lam Leung

**Affiliations:** 1 Family Care Physio Clinic, Singapore 127371, Singapore; 2 Department of Paediatrics & Adolescent Medicine, School of Clinical Medicine, LKS Faculty of Medicine, The University of Hong Kong, Pokfulam, Hong Kong; fkl117@hku.hk; 3 Cluster of Health and Social Sciences, Singapore Institute of Technology, 1 Punggol Coast Road, Singapore 828608, Singapore; 4 Department of Rheumatology, Allergy and Immunology, Tan Tock Seng Hospital, Singapore 308433, Singapore

**Keywords:** musculoskeletal pain, acupuncture, inflammation, cytokines

## Abstract

Background: Musculoskeletal pain remains a major cause of disability worldwide, encompassing disorders such as rheumatoid arthritis (RA), osteoarthritis (OA) and chronic back pain. Acupuncture and dry needling are increasingly used for symptom management, yet their effects on inflammatory modulation remain unclear. This systematic review and meta-analysis evaluated the influence of acupuncture on inflammatory biomarker regulation in musculoskeletal pain. Methods: Following PRISMA and Cochrane methodological guidelines, comprehensive searches were conducted across MEDLINE (via PubMed), Web of Science, Cochrane Library, Scopus, Google Scholar, and OpenEvidence from inception to August 2025. Eligible studies were randomized controlled trials (RCTs) involving acupuncture or dry needling interventions with inflammatory biomarker outcomes. Screening, data extraction, and risk of bias assessment using ROB2 were performed by two reviewers independently. The certainty of evidence was appraised using GRADE criteria. The protocol was registered on PROSPERO (CRD420251011831). Results: Nineteen RCTs and one randomized cross-over study (*n* = 1492) met inclusion criteria. Some studies demonstrated reductions in CRP, ESR, IL-1β, IL-6 and TNF-α following acupuncture. Random-effects meta-analysis indicated that modified acupuncture (electroacupuncture or needle-knife therapy) significantly reduced TNF-α in knee OA compared with traditional acupuncture (SMD = −1.63, 95% CI −2.47 to −0.80, *p* < 0.01) but not IL-1β. However, no significant effects were observed from acupuncture versus sham acupuncture for CRP or ESR in patients with arthritis. However, the findings are limited by high heterogeneity and the small number of studies included in each meta-analysis. Conclusions: A moderate level of GRADE evidence suggests that modified acupuncture may be more effective than standard acupuncture in reducing TNF-α levels in patients with OA. Further high-quality biomarker-based RCTs are warranted to confirm these findings. This study received no external funding.

## 1. Introduction

Across all age ranges, musculoskeletal (MSK) pain, including chronic lower back pain, fibromyalgia, osteoarthritis (OA), rheumatoid arthritis (RA) and degenerative or inflammatory conditions, represents a major source of disability. It significantly reduces patients’ daily functioning and imposes substantial healthcare and economic burdens worldwide [1,2].

Acupuncture, which involves diverse interventions such as traditional Chinese needle acupuncture, dry needling, and electroacupuncture, is frequently used for the management of MSK disorders [3,4,5,6]. Evidence supporting the use of acupuncture and dry needling (DN) for MSK pain is mixed. Earlier systematic reviews reported the efficacy of DN and acupuncture as insignificant or non-superior to placebo [7]. Subsequent studies incorporating updated methodologies and a larger evidence pool have reported more favorable pain reduction effects for DN from 72 h to 24 weeks after treatment [8].

Several scientific mechanisms have been proposed to explain the effects of acupuncture and DN. Short-term activation of the sympathetic nervous system has been suggested and demonstrated by a significant increase in heart rate (20.6% vs 5.3%) [9] and a significant increase in mean pupil diameter after needling [10]. Activation of the sympathetic nervous system has been suggested to improve both local and distant mechanical hyperalgesia. De Meulemeester et al. (2022) attempted to investigate the effects of DN on surface electromyography activities, but the results were insignificant [11]. The application of DN to myofascial trigger points (MTrPs) may mitigate pain by reducing the excitability of the central nervous system (CNS) and dorsal horn neurons [12]. However, their proposal was largely extrapolated from conceptual models, animal investigations, and preliminary pilot studies with limited sample sizes, many of which were published more than a decade ago. Given that both acupuncture and DN may stimulate the sympathetic nervous system, the precise mechanism underlying any subsequent systemic physiological alterations remains unclear.

In clinical practice, the levels of inflammatory biomarkers can indicate systemic physiological changes, and they are highly relevant to clinical presentation. In patients with sciatica, moderate positive correlations for serum and biopsy (r = 0.63–0.65) pro-inflammatory cytokine tumor necrosis factor (TNF)-α with pain level have been reported by previous longitudinal studies [13]. In individuals with RA, C-reactive protein (CRP), a key acute-phase inflammatory marker, has been shown to correlate significantly with joint tenderness, swelling, and levels of disability (r = 0.46–0.80) [14]. For shoulder pain, interleukin (IL)-1β and IL-6 were positively correlated with pain intensity (VAS) and negatively correlated with shoulder function scores [15,16]. A negative association was observed between matrix metalloproteinase (MMP)-1 and ASES scores, while overexpression of vascular endothelial growth factor (VEGF) was correlated with greater pain (VAS), higher microvascular density, and advanced stages of rotator cuff disease [17].

Collectively, the physiological mechanisms of acupuncture and dry needling can be investigated through studies that assess alterations in inflammatory biomarker levels. This systematic review evaluates the available evidence on the effects of acupuncture and dry needling in musculoskeletal pain, with particular emphasis on inflammatory biomarker responses rather than condition-specific clinical outcomes.

## 2. Materials and Methods

### 2.1. Protocol and Registration

This systematic review was conducted in accordance with the guidelines of Preferred Reporting Items for Systematic Reviews and Meta-Analyses (PRISMA) 2020 and Cochrane [18,19]. The protocol was registered on PROSPERO with the reference number CRD420251011831. Ethical approval and informed consent were not required for this systematic review.

### 2.2. Search Strategy

Electronic database searches were performed across the Cochrane Library, MEDLINE (via PubMed), Scopus and Web of Science (detailed strategies provided in Appendix A), with additional screening conducted through Google Scholar and OpenEvidence using Boolean logic. Reference lists of relevant reviews and included studies were also hand-searched to identify further eligible articles. The search was restricted to English-language publications to enhance consistency and transparency and covered all records from database inception to 29 September 2025. Where required, corresponding authors were contacted to clarify study details or provide missing data.

### 2.3. Inclusion and Exclusion Criteria

This review included randomized controlled trials (RCTs) involving individuals with musculoskeletal pain conditions, such as arthritis, different joint pain, tendinopathy, neck pain, or back pain. Eligible interventions comprised acupuncture or dry needling administered by professionally trained practitioners. The primary outcomes of interest were inflammatory cytokines and related biomarkers, including CRP, erythrocyte sedimentation rate (ESR), ILs, MMPs, TNF-α and other inflammation-relevant markers.

Studies were excluded if either the intervention or the control group received additional treatments such as medication, diet, or other passive interventions that could confound the specific effects of acupuncture or dry needling. Non-penetrating variations in acupuncture and dry needling, such as laser acupuncture, acupressure massage, and electrical stimulation of acupoints, were also excluded. Studies involving co-treatment were considered acceptable only when both groups received identical adjunctive interventions so that the results could be attributed solely to acupuncture or dry needling. Studies on post-surgical subjects were also excluded, as it would be difficult to distinguish whether observed inflammatory responses were attributable to musculoskeletal pain or surgical effects. In addition, studies that did not report outcomes related to inflammatory biomarkers were not included.

Retrieved records were imported into the Rayyan platform (https://www.rayyan.ai/ (accessed on 5 October 2025)) for removal of duplicates and study screening. Screening was conducted by two reviewers independently for all articles against the inclusion and exclusion criteria, followed by methodological quality appraisal after selection. Discrepancies were resolved through discussion, with input from a third reviewer when necessary. The first author has a diploma in acupuncture, and he has all the necessary professional knowledge regarding this area.

### 2.4. Data Extraction

Data extraction was performed independently by the reviewers, including participant demographics, primary diagnosis, and applied inclusion and exclusion criteria. Detailed information on interventions was also collected, including the type of needling technique and the corresponding treatments used in the control groups. Key outcome measures and corresponding statistical data were also extracted. For studies with comparable outcome measures, data were pooled using Review Manager (RevMan, version 5.4; The Cochrane Collaboration, UK) and analyzed with standard meta-analytic methods.

### 2.5. Risk of Bias Evaluation and Evidence Synthesis

Methodological quality was appraised using the Cochrane risk-of-bias tool for randomized trials version 2 (ROB2) for randomization, intervention adherence, and outcome reporting [20]. Evidence certainty was rated via the Grades of Recommendation, Assessment, Development, and Evaluation (GRADE) approach [21].

## 3. Results

**Figure 1:**
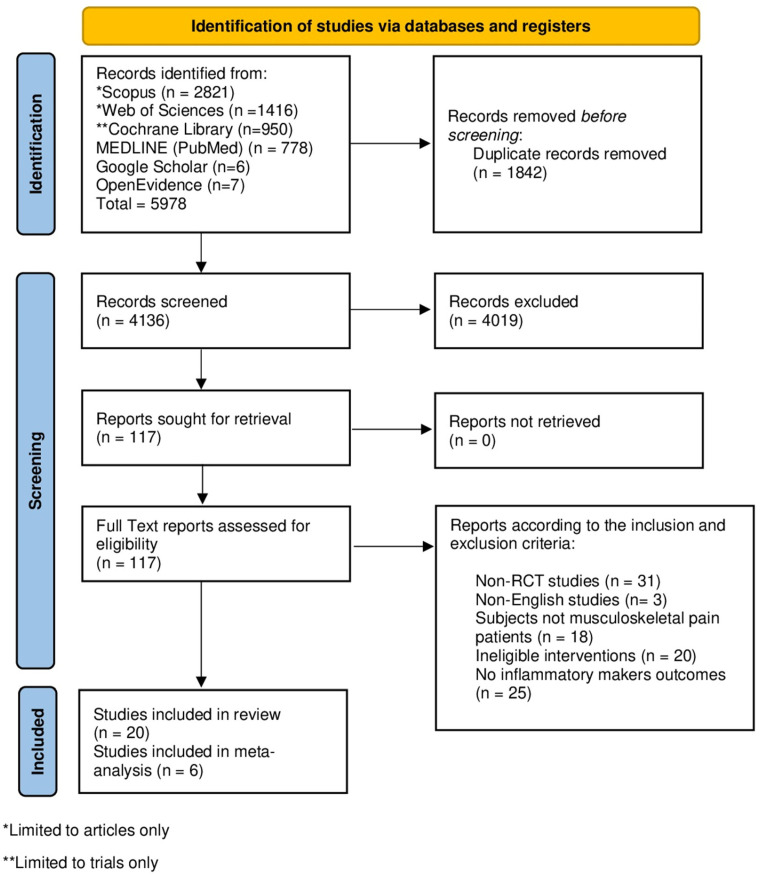
PRISMA Flowchart.

The database search yielded 5978 records. After removal of duplicates, 4136 unique studies remained for screening. Of these, 4019 were excluded during title and abstract review, primarily due to inappropriate study design (e.g., protocols, reviews or non-RCTs), non-musculoskeletal populations, or interventions not related to acupuncture or dry needling. A total of 117 full-text articles were reviewed in detail, and 19 RCTs and one cross-over study [22,23,24,25,26,27,28,29,30,31,32,33,34,35,36,37,38,39,40,41] ultimately met the inclusion criteria (Figure 1). In addition to duplication removal assisted by the Rayyan website, the screening and selection of studies were performed manually.

The included studies comprised 1492 participants, with ages ranging from 18 to 82 years. Across trials, the mean participant age was generally within the fourth to sixth decades of life. Detailed study characteristics are presented in Supplementary Tables S1 and S2. Of the 20 included studies, 11 investigated patients with knee osteoarthritis (KOA), five focused on RA, two focused on low back pain (one on lumbar disk herniation), and the remainder focused on acute gouty arthritis and chondromalacia patellae.

Regarding the experimental interventions, eight studies employed electroacupuncture, six studies applied manual acupuncture, three studies used warm needling with moxibustion, and the other studies included needle-knife therapy, auricular acupuncture, and triple needling combined with herbal decoction. The compared interventions also varied across the studies. Sham acupuncture was the most frequently used treatment. Conventional pharmacological treatments were used as controls in four studies, while standard care or adjunctive therapies appeared in two studies. In five trials, alternative acupuncture served as active comparators, such as manual versus electroacupuncture, or routine acupuncture versus specific needling techniques.

A wide range of inflammatory biomarkers was evaluated across the included trials. The most commonly measured cytokines were IL-1β and TNF-α, followed by IL-6. Several trials assessed systemic inflammatory indicators, such as CRP and ESR, while two studies measured rheumatoid factor, an autoimmune biomarker associated with RA. A smaller number of trials examined broader immunological mediators, including NF-κB, MMP-1, MMP-3, MMP-13, COMP (Cartilage oligomeric matrix protein), PL (Phospholipase)A2, α1-AGP (Alpha-1-acid glycoprotein), and sTNF-R1 (Soluble tumor necrosis factor receptor 1), as well as neuroinflammatory substances, such as substance P, prostaglandin E2, dopamine, and serotonin.

Several studies have reported other biological mediators in addition to inflammatory biomarkers. Neuroendocrine markers, such as cortisol and β-endorphin, have been assessed in multiple studies, along with growth and repair factors (e.g., TGF-β, IGF-1, FGF-2). A subset of studies explored oxidative stress markers (Superoxide dismutase (SOD) and MDA) and cartilage metabolism markers (COMP and MMPs). Other biochemical indices, such as uric acid, α1-AGP, and general blood chemistry, were also measured. Most trials also reported other clinical outcomes, including pain intensity (VAS), functional disability indices (e.g., WOMAC, Lequesne), and quality of life measures (e.g., HAQ, EuroQol, and GHQ-28). These non-inflammatory markers and clinical outcomes were recorded but were not included in the synthesis.

Among the included studies, many reported significant within-group reductions in inflammatory biomarkers, such as IL-1β, IL-6, TNF-α, CRP, and ESR, following acupuncture. However, only a subset of trials demonstrated a significant between-group difference compared with control interventions. Four trials [31,33,39,40] found no significant between-group differences in inflammatory outcomes.

### 3.1. Risk of Bias Assessment

**Figure 2:**
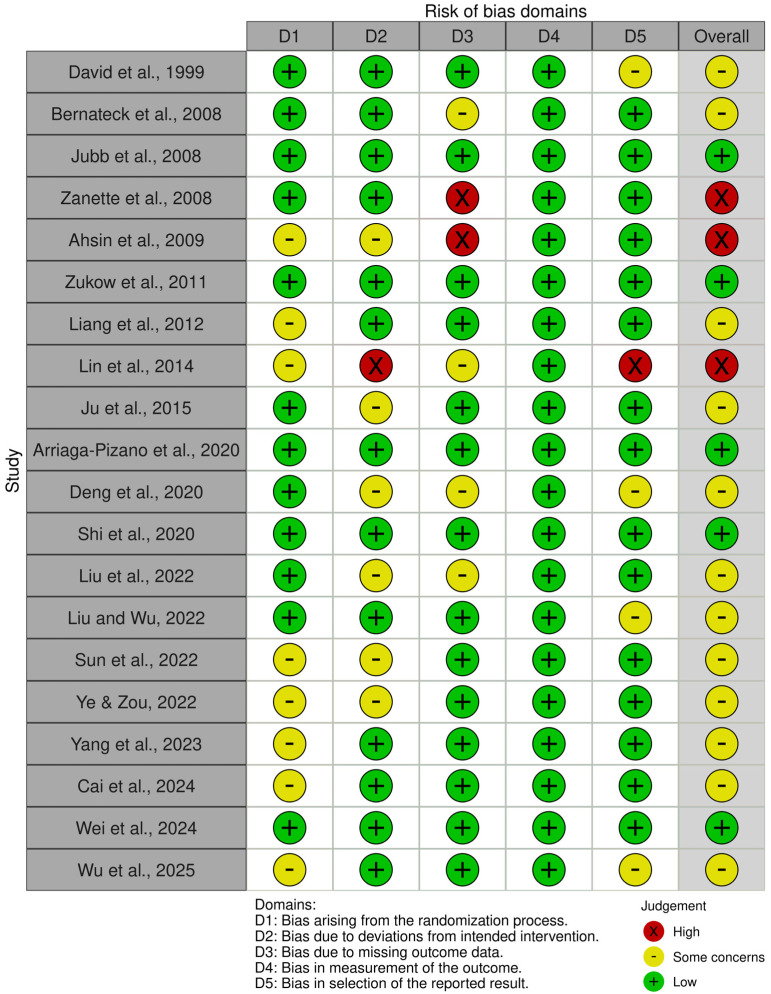
Risk of bias assessment.

In this review, the risk of bias assessment was limited to biomarker outcomes and excluded other clinical measures. This focused evaluation could have influenced the overall bias profile, as the inclusion of additional clinical endpoints might have led to different judgments of study quality. According to the ROB2 assessment, five studies were judged as having a low risk of bias, 12 as having some concerns, and three as having a high risk of bias (Figure 2).

**Figure 3:**
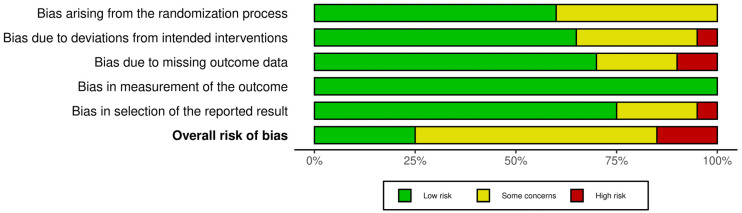
Risk of bias summary.

Concerns were most pronounced in the domains of deviations from intended interventions (D2) and missing outcome data (D3), where several studies demonstrated either a high risk or some concerns. The randomization process (D1) and issues related to selective reporting (D5) were generally low risk in most of the studies. The measurement of outcomes (D4) was consistently judged to be low risk across all included trials because this review only focused on inflammatory biomarkers as outcome measures (Figure 3).

Three trials were judged to have a high risk of bias. The study by Lin et al. (2014) [22] was rated as high-risk because, although 85 participants were initially assigned to each group, only 76 and 75 participants in the treatment and control groups, respectively, were included in the analysis. The excluded participants were not accounted for when using an intention-to-treat approach. In addition, this study selectively reported only part of the biomarker outcomes [22]. The studies by Ahsin et al. (2009) and Zanette et al. (2008) were listed as high risk, particularly in bias due to missing outcome data (D3), simply because of the remarkably high dropout rate of their subjects [36,39].

### 3.2. Meta-Analysis

Four sets of meta-analyses were performed from six studies, grouped according to shared population characteristics, interventions, comparators, and inflammatory biomarker outcomes. A random-effects model was used for data synthesis. Mean differences (MD) were calculated for outcomes measured on the same scale, whereas standardized mean differences (SMD) were applied when different measurement scales were used. All estimates were reported with 95% confidence intervals (CIs), and statistical heterogeneity was assessed using the I^2^ statistic.

**Figure 4:**
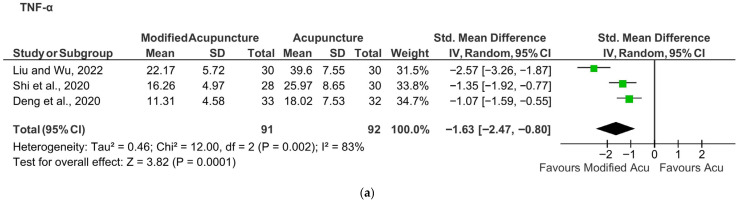

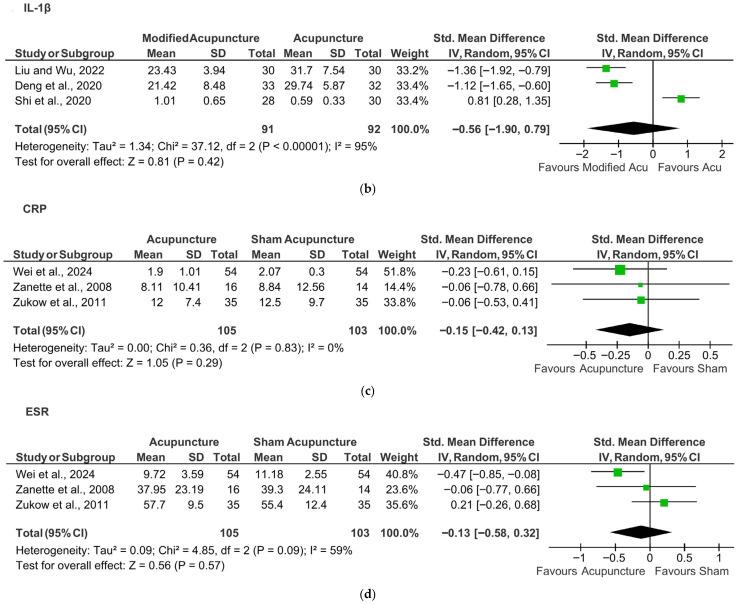
(**a**) TNF-modified vs standard acupuncture in KOA. (**b**) IL-1-modified vs standard acupuncture in KOA. (**c**) CRP acupuncture vs sham acupuncture in arthritis. (**d**) ESR acupuncture vs sham acupuncture in arthritis.

From three included studies with KOA patients 29,30,38, modified acupuncture was found to be significantly more effective in reducing TNF-α than regular acupuncture (SMD −1.63; 95% CI: −2.47 to −0.8, *p* < 0.01, I^2^ = 83%) (Figure 4a), but not for IL-1β (SMD −0.56; 95% CI: −1.90 to 0.79, *p* = 0.42, I^2^ = 95%) (Figure 4b). Modified acupuncture included electroacupuncture and needle-knife therapy, which had different or additional stimulation with the same traditional meridian-based point selection as the comparison group.

A meta-analysis of three other studies on patients with arthritis, namely OA and RA [23,33,39], showed that acupuncture had no significant effects on CRP and ESR compared with sham acupuncture (Figure 4c,d).

### 3.3. GRADE Recommendation

Based on the meta-analysis findings, moderate-certainty evidence (GRADE) indicates that modified acupuncture is more effective than standard acupuncture in reducing TNF-α levels in patients with KOA (Table 1). However, this finding should be interpreted with caution because of the high level of heterogeneity (I^2^ = 83%).

## 4. Discussion

In our systematic review, the findings showed that modified acupuncture is more effective than regular acupuncture in controlling TNF-α in patients with KOA. On the other hand, the effects of acupuncture on CRP and ESR are not significantly different from those of sham acupuncture in patients with arthritis.

In the comparison between modified acupuncture and standard acupuncture in patients with KOA, the pooled SMD for TNF-α was 1.63 with an overall effect Z =3.82 and heterogeneity (I^2^ = 83%). Standardized mean differences of approximately 0.2, 0.5, and 0.8 are typically considered small, moderate, and large effects, respectively [42]. A Z value of 1.96 or greater corresponds to *p* < 0.05 for a two-tailed test, indicating statistical significance at the conventional threshold [43]. However, the high heterogeneity (I^2^ = 83%) suggests that there are remarkable methodological differences between the included studies [44,45]. In this case, it is the major reason for downgrading the GRADE recommendation (Table 1), and caution should be taken when interpreting the meta-analysis results. 

A potential source of the high heterogeneity observed in this review lies in differences in study inclusion criteria and clinical characteristics across the three RCTs [29,30,38]. Although all studies focused on patients with KOA, in the inclusion criteria, there were variations in age, disease severity and symptom duration. In addition, two studies collected biomarkers through serum [29,30] and one through joint fluid [38]. These are the possible methodological factors contributing to the high heterogeneity. Despite these factors, the included studies shared key clinical characteristics, including a common target population and broadly comparable acupuncture-based interventions. Therefore, the pooling of data based on similar PICO elements remains methodologically and clinically justifiable. In the studies, both modified acupuncture and standard acupuncture groups demonstrated significant within-group reductions in inflammatory markers compared to baseline. A key limitation is the lack of direct comparisons with placebo or sham controls within these studies, which limits the ability to determine whether modified acupuncture or standard acupuncture is superior to control or placebo intervention.

A previous systematic review and meta-analysis by Lin et al. (2016) [46] reported that acupuncture produced significant short-term improvements (up to 13 weeks) in both pain (*p* < 0.01; WMD = −1.24 [95% CI, −1.92 to −0.56]; I^2^ > 50%) and physical function (*p* < 0.01; WMD = 4.61 [95% CI, 2.24 to 6.97]; I^2^ > 50%) in the short term (up to 13 weeks) [46]. A systematic review by Liu et al. (2021) [47] concluded that fire needles, warm needles, and electroacupuncture were more effective than regular acupuncture and Western medicine in improving joint function scores and pain severity [47]. These results align with those of the present review. 

Regarding the biomarkers, the levels of TNF-α in synovial fluid correlate with movement and resting pain in patients with OA, and elevated TNF-α was also found in low- and high-grade synovitis, reflecting ongoing inflammatory activation in late-stage OA [48]. Synovial fluid TNF-α was shown to have a statistically significant but weak correlation with pain scores (r = 0.25–0.29, *p* < 0.05) [49,50]. Taken together, these findings may suggest the possible effectiveness of acupuncture in regulating inflammatory pain in patients with OA. 

Pro-inflammatory cytokine IL-1β in OA contributes to cartilage degradation through the induction of matrix-degrading enzymes and inhibition of anabolic repair processes [51,52]. Synovial fluid IL-1β showed modest negative correlations with pain intensity (r = −0.28 to −0.20); it was also negatively correlated with K–L grade (r = −0.363) and overall WOMAC score (r = −0.317) [50]. Based on the meta-analysis in our review, no significant difference but substantial heterogeneity (I^2^ = 95%) was observed between modified acupuncture and standard acupuncture in reducing IL-1β levels.

The CRP and ESR levels are typically elevated in RA, reflecting the systemic inflammatory nature, but remain normal or only mildly elevated in OA [50,53,54]. From synovial biopsy in patients with RA, CRP had a moderately strong correlation with histological inflammation (rho = 0.43, *p* < 0.01), while ESR showed a weaker correlation (rho = 0.29, *p* < 0.01) [55]. Emerging evidence shows that OA also has low-grade systemic inflammation, and ESR and CRP are usually within the normal range [54]. However, in OA cases with elevated CRP and ESR, the elevation is modest and reflects the indolent inflammatory component of OA rather than robust systemic inflammation [56,57]. Elevated high-sensitivity CRP, but not ESR, correlates with symptoms (pain and physical function) rather than radiographic severity [53,58]. After all, meta-analysis of three RCTs in this review showed no significant effect of acupuncture on CRP and ESR levels in patients with arthritis (RA and OA), with low to moderate heterogeneity (I^2^ = 0% and 59%).

It should be noted that this review focused specifically on inflammatory biomarkers and did not include direct analysis of clinical outcomes such as pain, disability, or functional status. While these outcomes are clinically important, they represent distinct domains that may not necessarily correlate with changes in individual inflammatory markers. In addition, such outcomes are often subjective and may be influenced by different sources of bias than those affecting biochemical measures.

All included trials employed acupuncture techniques based on TCM theory, despite notable heterogeneity in acupoint selection and intervention techniques. The interventions consistently followed meridian-based point selection (e.g., ST36, SP9, LI4, GB34, and LV3), with needle manipulation aimed at eliciting the de qi sensation (specific feeling responding to TCM acupuncture treatment). None of the studies described the use of anatomical trigger-point localization, segmental neurophysiological rationale, or Western medical acupuncture framework. Similarly, no protocol identified the intervention as dry needling, and no trials adopted a myofascial or motor-point-targeted methodology. TCM orientation was further evidenced by the frequent use of adjunctive techniques (e.g., warm needling, auricular acupuncture, or needle-knife therapy) and theoretical references to concepts such as qi regulation and Bi-syndrome [27,39,40]. A possible reason is that available RCTs on dry needling mostly applied clinical outcomes, such as pain and disability measurements [8,59], and available biochemical studies on the effects of dry needling are mostly in animals [60,61]. Therefore, clinical trials with dry needling interventions could not fulfill the inclusion criteria for this systematic review.

A recent systematic review reported that isokinetic exercise significantly reduced CRP levels in patients with OA and back pain compared with general exercise (mean difference: −0.40; 95% CI: −0.44 to −0.36; *p* < 0.01; I^2^ = 0%), with high-certainty evidence based on GRADE [62]. In contrast, GRADE evidence for its effects on TNF-α and IL-6 was rated low, and the certainty of osteoarthritis-specific outcomes was very low. Therefore, it may be worth exploring the effects of combining acupuncture and exercise in clinical practice to control inflammation in patients with OA.

In addition to OA and musculoskeletal pain, the effects of acupuncture on inflammatory responses have been studied under other conditions. For example, in cancer patients, acupuncture has been shown to be effective in reducing the levels of IL-1, IL-4, IL-6 and CRP, but not significantly in reducing white blood cell count, IL-2, IL-10, or TNF-α (Liu et al., 2024) [63]. According to the systematic review by Lu et al. (2022) [64], combining acupuncture and Western medicine is significantly more effective than Western medicine alone in reducing CRP (weighted mean difference [WMD]: −6.30; 95% CI: −9.08, −3.52) and ESR (WMD: −6.56; 95% CI: −8.60, −4.52) in RA patients [64].

From a clinical perspective, the management of OA is primarily non-pharmacological, focusing on exercise, weight reduction (when indicated), and patient education, while pharmacological therapies are mainly used as adjuncts for symptom control rather than as primary treatments [65]. Although TNF-α is implicated in OA pathophysiology, pharmacological clinical trials targeting TNF-α have consistently failed to demonstrate meaningful therapeutic benefit for OA management [65,66]. The present meta-analysis demonstrated that modified acupuncture may influence TNF-α levels, and previous systematic reviews have reported beneficial effects of acupuncture on OA-related symptoms [46,47]. Consequently, the clinical effects of acupuncture are unlikely to be mediated through a single inflammatory pathway and should instead be understood within a broader, multimodal framework, and TNF-α may be a better objective indicator to reflect the treatment response than CRP and ESR in OA.

### Limitations

A significant proportion of acupuncture-related RCTs are published in Chinese. However, this systematic review was restricted to English-language publications to ensure consistency in data extraction and quality appraisal. Of the 20 included studies, the majority (i.e., 13 RCTs) were conducted in China, reflecting the regional concentration of research in this field. This language restriction may have limited the comprehensiveness of the evidence base and introduced potential language and regional bias.

If no language restriction had been applied, a substantial proportion of additional eligible studies would likely have been published in Asian languages, particularly Chinese, Japanese, and Korean. While inclusion of these studies may have improved the comprehensiveness of the evidence base, it would also have introduced practical challenges related to translation, data extraction consistency, and quality appraisal across different languages. Therefore, the language restriction represents a balance between comprehensiveness and methodological feasibility but may have contributed to potential language bias.

Due to the small number of studies included in each meta-analysis, formal assessment of publication bias, such as funnel plots or statistical tests for small-study effects, was not performed. Meta-analysis in this review involved fewer than 10 studies; the power of both visual inspection and statistical tests (Begg and Egger tests) to detect publication bias is far less than 80%, making these approaches of limited value (Jassim et al. 2023; Rao et al. 2017) [67,68]. In fact, because 90% of meta-analyses have ≤10 studies, funnel plot approaches are underpowered (Rao et al. 2017) [68]. With three studies, most statistical methods can satisfy nominal coverage probability, which is an improvement over two-study meta-analyses where no method performs well in random effects scenarios [69]. Although sensitivity analyses can be conducted for meta-analysis with three studies, the removal of individual studies could result in substantial changes in both the magnitude and direction of the effect in our meta-analysis. This reflects the limited number of included studies and the high degree of heterogeneity, indicating that the findings are not robust and should be interpreted with caution.

Another limitation of this review is the heterogeneity of the study populations. Although the review aimed to evaluate the effects of acupuncture in individuals with generalized musculoskeletal pain, most included participants were diagnosed with OA. Consequently, the review is deficient in evidence pertaining to other common MSK conditions such as tendinopathy, neck pain, and shoulder pain.

In data extraction, the biological source of inflammatory biomarkers (e.g., serum, plasma, synovial fluid) was extracted and recorded. However, the reporting of sampling compartments was inconsistent across the included trials. For several cytokines, the number of contributing trials was small, often limited to two or three studies per biomarker. This restricts the robustness of the pooled estimates and contributes to the observed heterogeneity. Therefore, these results warrant cautious interpretation, particularly for TNF-α outcomes with high I^2^ values.

## 5. Conclusions

This systematic review provides an updated summary of the effects of acupuncture on the regulation of inflammatory markers in patients with MSK pain. Specific moderate GRADE recommendations suggest modified acupuncture’s potential to down-regulate TNF-α in KOA patients. However, the findings should be interpreted with caution due to heterogeneity and the limited number of contributing trials. These findings provide additional insights supporting the hypothesis that regulation of the immune system constitutes one of the therapeutic mechanisms underlying acupuncture interventions.

## Figures and Tables

**Table 1:** GRADE recommendation on modified acupuncture compared to acupuncture for KOA on TNF-α.

Certainty Assessment							№ of Patients		Effect	Certainty	Importance
№ of Studies	Study Design	Risk of Bias	Inconsistency	Indirectness	Imprecision	Other Considerations	Modified Acupuncture	Acupuncture	Absolute (95% CI)		
TNF-α (follow-up: 4 to 21 weeks)											
3	RCT	not serious *a	Serious *b	not serious	not serious	none	91	92	SMD 1.63 SD lower (2.47 lower to 0.80 lower)	⨁⨁⨁◯ Moderate *a,b	IMPORTANT

RCT: randomized controlled trial; CI: confidence interval; SMD: standardized mean difference; ⊕: level of certainty. *a. According to ROB2 assessment, two articles have some concerns about overall bias, and one article has a low risk of bias; potential limitations are unlikely to lower confidence in the estimate of effect. *b. Heterogeneity I^2^ = 83%, Z = 3.82; *p* < 0.01.

## Data Availability

No new data were created or analyzed in this study.

## Supplementary Materials

The following supporting information can be downloaded at: https://www.mdpi.com/article/10.3390/muscles5020036/s1, Supplementary Table S1: Characteristics of included studies; Supplementary Table S2: Outcome measures and follow-up periods of the included studies.

## Abbreviations

The following abbreviations are used in this manuscript:

CI	Confidence interval
CNS	Central nervous system
COMP	Cartilage oligomeric matrix protein
CRP	C-reactive protein
DN	Dry needling
EA	Electroacupuncture
ESR	Erythrocyte sedimentation rate
EuroQol	EuroQol quality of life scale
GHQ-28	General Health Questionnaire-28
GRADE	Grading of Recommendations, Assessment, Development and Evaluation
HAQ	Health Assessment Questionnaire
I^2^	Heterogeneity statistic (I-squared)
IL	Interleukin
KOA	Knee osteoarthritis
MA	Manual acupuncture
MD	Mean difference
MDA	Malondialdehyde
MMP	Matrix metalloproteinase
MSK	Musculoskeletal
NF-κB	Nuclear factor kappa B
OA	Osteoarthritis
PICO	Population, Intervention, Comparison, Outcome
PLA2	Phospholipase A2
PROSPERO	International Prospective Register of Systematic Reviews
PRISMA	Preferred Reporting Items for Systematic Reviews and Meta-Analyses
RA	Rheumatoid arthritis
RCT	Randomized controlled trial
ROB2	Risk of bias tool version 2
SMD	Standardized mean difference
SOD	Superoxide dismutase
TCM	Traditional Chinese Medicine
TNF-α	Tumor necrosis factor alpha
VAS	Visual analog scale
WOMAC	Western Ontario and McMaster Universities Osteoarthritis Index

## Appendix A. Search Strings and Databases

Scopus

TITLE-ABS-KEY (pain*)

AND TITLE-ABS-KEY(hip* OR knee* OR ankle* OR shoulder* OR wrist* OR hand*

OR spine* OR back OR neck* OR arthrit* OR musculoskeletal* OR limb* OR tendon*)

AND TITLE-ABS-KEY(acupunctur* OR electroacupunctur* OR “acupuncture therap*”

OR “dry needl*” OR needl* OR “acu-point*” OR “acu point*”)

AND TITLE-ABS-KEY(“C-reactive protein*” OR interleukin* OR “tumor necrosis factor*”

OR biomarker* OR “inflammator* marker*” OR “biological marker*”

OR inflammat* OR cytokin* OR serum OR plasma OR saliva*)

Web of Science (WOS)

#1: (“pain*”)

#2: (“hip*” OR “knee*” OR “ankle*” OR “shoulder*” OR “wrist*” OR “hand*”

OR “spine*” OR “back” OR “neck*” OR “arthrit*” OR “musculoskeletal*”

OR “limb*” OR “tendon*”)

#3: (“acupunctur*” OR “electroacupunctur*” OR “acupuncture therap*”

OR “dry needl*” OR “needl*” OR “acu-point*” OR “acu point*”)

#4: (“C-reactive protein*” OR “interleukin*” OR “tumor necrosis factor*”

OR “biomarker*” OR “inflammator* marker*” OR “biological marker*”

OR “inflammat*” OR “cytokin*” OR “serum” OR “plasma” OR “saliva*”)

#1 AND #2 AND #3 AND #4

Limited to articles only

Cochrane Library

pain AND (hip OR knee OR ankle OR shoulder OR wrist OR hand OR spine OR back

OR neck OR arthritis OR MSK OR musculoskeletal OR limb* OR tendon*)

AND (acupunctur* OR electroacupunctur* OR acupuncture NEXT therap*

OR dry NEXT needl* OR needl* OR acu-point* OR acu NEXT point*)

AND (C-reactive NEXT protein* OR interleukin* OR tumor NEXT necrosis NEXT factor*

OR biomarker* OR inflammator* NEXT marker* OR biological NEXT marker*

OR pro-inflammator* NEXT marker* OR inflammat* OR cytokin*

OR serum OR plasma OR saliva*)

Limit to: Trials only

MEDLINE (via PubMed)

((pain) AND ((Hip) OR (Knee) OR (Ankle) OR (Shoulder) OR (Wrist) OR (Hand)

OR (Spine) OR (Back) OR (Neck) OR (Arthritis) OR (Musculoskeletal)

OR (Limb) OR (Tendon)))

AND

((“Acupuncture”[MeSH]) OR (“Electroacupuncture”[MeSH]) OR (“Acupuncture Therapy”[MeSH])

OR (Acupuncture) OR (Electroacupuncture) OR (“Dry needling”) OR (Needling)

OR (“Acu-point”) OR (“Acu point”)))

AND

((“C-reactive protein”[MeSH]) OR (Interleukin) OR (Tumor Necrosis Factor)

OR (“Biomarkers”[MeSH]) OR (“Inflammatory marker”) OR (“Biological marker”)

OR (“Inflammation”[MeSH]) OR (“Cytokines”[MeSH]) OR (Serum) OR (Plasma) OR (Saliva)))
