# Supplementary material for: Immunomodulatory Effects of Acupuncture on Inflammatory Markers in Patients with Musculoskeletal Pain: A Systematic Review of Randomized Controlled Trials
Source: Muscles. 2026 May 8;5(2):36. doi: 10.3390/muscles5020036 (PMC13214626; doi:10.3390/muscles5020036)
Supplement: Supplementary file 1 [file muscles-05-00036-s001.zip › Supplementary Table S2.pdf]

Supplementary Table S2. Outcome measures and follow-up periods of the included studies

		Intervention group	Comparison 1	Outcome measures		Follow up period	Major results
S/N	Authors			Inflammatory biomarkers	Non-inflammatory biomarkers and Other outcomes		
1	David et al., 1999	Acupuncture at single point Liver 3 (bilateral), inserted for 4 min with manual manipulation at 2 min for 5s; once weekly × 5 sessions	Placebo acupuncture (needle introducer held without skin penetration at Liver 3 for 4 min, identical setting)	ESR, CRP	VAS pain, VAS patient global, 28 swollen joint count, 28 tender joint count, analgesic use, GHQ-28 (subscales A–D), Modified DAS index	Baseline week 1, post-treatment (week 6) second baseline (week 12), post-second treatment (week 17) and 6 weeks post-treatment (week 22)	No significant treatment, period, or interaction effects for any outcome; no difference between acupuncture and placebo
2	Bernateck et al., 2008	Auricular electroacupuncture + autogenic training; 20 min/session, 2×/week × 10 weeks	Autogenic training alone	ESR, CRP	Pain VAS, HAQ, morning stiffness	Post-treatment (10 weeks) and follow-up at 3 months	Both groups showed decreased ESR and CRP, but reductions were more pronounced in the auricular EA group
3	Jubb et al., 2008	Manual + electroacupuncture at LI4, SP10, Xiyan, SP9, GB34, ST36, LIV3, BL40, BL57; 2x/week × 5 weeks	Sham acupuncture using non-penetrating needles + sham electroacupuncture (same points, dummy mode)	Plasma β-endorphin	WOMAC (pain, stiffness, function); VAS pain (night, weight-bearing, general); EuroQol score	Baseline, end of 5-week treatment, and 1-month post-treatment	Plasma β-endorphin between group analysis at the three time points gave no significant difference.
4	Zanette et al., 2008	Standard acupuncture (10 sessions over 5 weeks)<br>Acu points: EX1, PC6, LI4, EX28, CV12, CV6, ST36, SP6, LV3, UB20, UB22, UB23, GV4, GV14, UB11, UB60	Sham acupuncture (superficial insertion, non-acupoints, no "teh Qi")	ESR, CRP	ACR20, DAS, HAQ, VAS pain, morning stiffness, tender/swollen joint count, global assessments by physician and patient	Visit 2: after 5th session; Visit 3: after 10th session; Visit 4: 1-month post-treatment	No significant difference in ESR/CRP between groups

5	Ahsin et al., 2009	Electro-acupuncture at ST35, EX-LE4, GB34, SP9, SP10, ST36, LR3; 20–25 min/session daily × 10 days	Sham acupuncture (superficial insertion at non-acupoints, no current)	Plasma cortisol	Plasma β-endorphin; WOMAC index; VAS pain; NSAID consumption	Post-treatment (10 days) and follow-up at 12 weeks	EA group had greater fall in plasma cortisol ($p=0.016$) vs sham
6	Zukow et al., 2011	Acupuncture at local, regional, distant and auricular points; 36 sessions (3×12), 2×/week	Sham acupuncture (non-acupoints, at least 5 cm from classical points, same treatment duration and needle type)	ESR, CRP, RF	VAS (pain), Modified Laitinen and Zytkowski questionnaire (pain intensity, frequency, analgesic use, mobility), X-ray erosion	Pre- and post-treatment (approx. 18 weeks)	No significant difference in ESR, CRP or RF
7	Liang et al., 2012	Acupuncture (BL20, BL23, ST36, CV4 + local/Ashi points: Ex-LE10, LI5, PC7, LI11, TE10, LI15, TE14, Ex-LE5, ST41, Ex-UE9); even reinforcing-reducing manipulation, 30 min, 5×/week, 30 days per course × 3 courses + Meloxicam 75 mg daily + Methotrexate 10 mg weekly + Leflunomide 10 mg daily	Same medication protocol without acupuncture	ESR, CRP, RF	HAQ score; Morning stiffness time; Average grip strength; Joint tenderness; Joint swelling; ADL	Baseline and 3 months post-treatment	Both groups improved significantly in ESR, CRP. Observation group had significantly greater improvement in CRP reduction vs control<br><br>RF decreased in both groups, but no between group difference.
8	Lin et al., 2014	Needle-knife therapy at Neixiyan, Waixiyan, GB39, SP10, ST35, KI3; 2 courses, 6-day interval	Routine acupuncture at same points; 2 courses, daily for 5 days with 2-day interval	Synovial fluid IL-1β, IL-6, TNF-α (ELISA)	Total symptom score; Clinical effectiveness (excellent/good/moderate/bad); Symptom scores, clinical effectiveness rates, X-ray; Adverse events	Post-treatment only<br><br>About 2 weeks	Treatment group showed greater symptom improvement in stages III & IV; significant more decrements in IL-1β, IL-6, TNF-α than control

9	Ju et al., 2015	High intensity Electro-acupuncture at EX-LE5, ST35, EX-LE4, GB34, ST36, SP9;<br>30 min/session, 3×/week for 8 weeks	Low intensity Electro-acupuncture	Serum IL-1β, TNF-α, IL-6	WOMAC index; VAS pain	Baseline and 8 weeks	EA group showed significant decreases in IL-1β, TNF-α, IL-6 (all p<0.01) vs control
10	Arriaga-Pizano et al., 2020	Electroacupuncture (EA) at ST36, BL25, GB30, BL40, GB34, 15 min/day for 5 days	Sham EA (non-points, superficial, no current); Diclofenac sodium (100 mg BID × 5 days)	TNF-α (in LPS-stimulated blood samples); Epinephrine and norepinephrine levels measured in serum	Serum biochemistry: Glucose, Urea, Creatinine, Uric Acid, Hemoglobin, Hematocrit, RBC indices (MCV, MCH, MCHC), White blood cell count, Platelets	5 treatment sessions; blood tested at baseline, after 1st and 5th sessions	EA increased epinephrine but not norepinephrine or dopamine. In vitro, epinephrine and norepinephrine (but not dopamine) suppressed LPS-induced TNF-α production.
11	Deng et al., 2020	Stuck-needle technique at ST35, EX-LE4, ST36, GB34, SP9, SP10, ST34, EX-LE2; daily ×14 days/course, 2 courses total	Regular acupuncture at same points, same course	IL-1β, TNF-α (joint fluid, ELISA)	VAS, Lequesne index, total effective rate, adverse events	Pre- and post-treatment (approx. 4 weeks)	Both groups improved significantly in VAS, Lequesne, IL-1β, TNF-α (P<0.05); stuck-needle group had greater reductions in all outcomes vs control (P<0.05).
12	Shi et al., 2020	Electro-acupuncture (EA) at local points [ST34, ST35, ST36, Heding, Neixiyan, GB33, GB34, SP9, SP10, LR7, LR8, Ashi] and distal points [GB31, GB36, GB39, GB41, ST40, ST41, LR3, BL60, SP6, KI3]; 24 sessions, 30 min each, over 8 weeks (3/week)	Manual acupuncture (MA) at same points, same schedule, no electrical current	TNF-α, IL-1β, IL-8, IL-18, MCP-1, IL-13, CCL-5, COMP, MMP-1, MMP-3, MMP-13	WOMAC (pain, stiffness, function), VAS pain, response rate (≥50% WOMAC improvement), adverse events	Baseline and post-treatment (8 weeks)	Both EA and MA significantly reduced TNF-α, IL-1β, MMP-3, MMP-13 and increased IL-13; EA showed greater TNF-α reduction than MA (p=0.046) but no other between-group cytokine differences

13	Liu et al., 2022	Electroacupuncture (Ashi, SP2, LR3, SP3, ST44, SP6, ST36, SP9; 30 min, every 3 days × 3 sessions) + Diclofenac sodium 50 mg/day × 7 days	Low-dose DS: Diclofenac sodium 50 mg/day × 7 days;<br><br>Conventional DS: Diclofenac sodium 100 mg/day × 7 days	CRP, WBC, NE%	VAS, joint tenderness, joint swelling, activity limitation, GIS (5 domains), SUA; adverse events	Pre- and post-treatment (7 days)	CRP decreased in all groups but no between-group difference)<br><br>WBC and NE% decreased significantly in EA + DS vs low-dose DS ($P < 0.05$)<br><br>SUA reduced only in EA + DS
14	Liu & Wu, 2022	Electroacupuncture (EA) at ST34, SP10, ST35, EX-LE4, GB34, EX-LE2, SP6; Dense wave 100 Hz; 30 min/day for 21 days	Comparison 1: Acupuncture (same points, no electric stimulation)<br><br>Comparison 2: Medication (Celecoxib 0.2g/day × 21 days)	IL-1β, TNF-α (serum)	WOMAC, VAS pain, Clinical effective rate	Pre- and post-treatment (21 days)	EA showed significantly greater reduction in IL-1β and TNF-α than acupuncture or medication.
15	Sun et al., 2022	Warm acupuncture + meloxicam + comprehensive nursing (4 weeks)	comparison 1: Meloxicam + comprehensive nursing<br><br>comparison 2: Warm acupuncture + comprehensive nursing	Serum PGE2, Substance P, DA, 5-HT (ELISA)	SOD, MDA<br>Knee function scores (pain, mobility, stability, walking ability, stairs); Clinical symptom improvement time (pain, swelling, movement limitation); Clinical efficacy rate (VAS); Oxidative stress indicators	Baseline, day 7, day 14, day 28	Combined group showed greater reductions in PGE2, SP, DA, 5-HT compared with control and TCM groups
16	Ye & Zou, 2022	Warm needling moxibustion at SP10, ST34, ST35, EX-LE4 (affected side), and bilateral SP6, LR3; 30 min/session, once/day, 5x/week for 5 weeks	Sodium hyaluronate injection (2 mL/week for 5 weeks)	NF-κB, TNF-α, IL-1β (knee joint fluid)	WOMAC, staircase time and VAS pain, MRI cartilage grading, clinical recovery rate (per TCM criteria)	Pre- and post-treatment (5 weeks)	Greater reduction in NF-κB, TNF-α, and IL-1β levels in the warm needling moxibustion group compared to the control group after 5 weeks

17	Yang et al., 2023	Acupuncture (9 EITBM points: GB39, BL60, KI13, ST34, SP10, SP9, GB34, EX-LE4, ST35) + standardized exercise; 25 min, 3×/week for 4 weeks	Acupuncture (6 classical points: ST34, SP10, EX-LE4, ST35, SP9, GB34) + same exercise	IL-1β, TNF-α, MMP-13 (ELISA, serum)	VAS, WOMAC (pain, stiffness, function), ROM, clinical effective rate	Pre- and post-treatment (4 weeks)	Both groups improved significantly; intervention group had greater reductions in IL-1β, TNF-α, MMP-13
18	Cai et al., 2024	Warming triple needling at EX-LE4, ST36, SP9, ST35 + Fang Feng Xi Bi Tang (herbal decoction); 3×/week for 4 weeks	Fang Feng Xi Bi Tang alone	IL-6, TNF-α, IL-1β (serum, ELISA)	TCM symptom score, WOMAC (pain, stiffness, function), clinical effectiveness	Pre- and post-treatment (4 weeks)	Significant reduction in IL-6, TNF-α, IL-1β in both groups; more improvement in acupuncture-med group ($P<0.05$)
19	Wei et al., 2024	Standard treatment (education, lifestyle, celecoxib 200mg/day) + acupuncture at EX-LE4, EX-LE5, GB34, ST36; daily, 6x/week for 4 weeks	Standard treatment + sham acupuncture (non-acupoints, shallow insertion, no Deqi, no manipulation)	ESR, CRP, IL-1 β, IL-6	TGF-β, IGF-1, FGF-2<br>Michel Lequesne index, VAS, LKSS, p38 MAPK mRNA	Post-treatment only (4 weeks)	Acupuncture group had larger reductions in ESR, CRP, IL-1β, IL-6; increased TGF-β, IGF-1, FGF-2; larger p38 MAPK mRNA reduction
20	Wu et al., 2025	Dragon-tiger fight needling method (GB30, BL37, BL60, BL57, GB31, GV3, BL54, BL25, Ashi); 30 min/session, every other day × 1 month + conventional Western treatment	Conventional Western treatment only	α1-AGP, PLA2, BK, 6-keto-PGE1α; sTNF-R1	↑ M-ENK, β-EP; SCV ↑ (sural & superficial peroneal nerves); ↓ lumbar flexion-extension ratio; ↑ torque & power<br><br>ODI; TCM symptom scores (cold pain, heaviness, pain at rest, difficulty turning, worse in cold);	Pre- and post- 1 month	Acupuncture significantly reduced pain and inflammatory markers<br><br>The method showed clinical superiority over control across all outcomes

Abbreviation Legend

ACR, American College of Rheumatology; ADL, Activities of Daily Living; Ashi, painful local points; BK, Bradykinin; BL, Bladder meridian; CCL-5, Chemokine (C-C motif) ligand 5; CLBP, Chronic Low Back Pain; COMP, Cartilage Oligomeric Matrix Protein; CRP, C-Reactive Protein; CV, Conception Vessel; DAS, Disease Activity Score; EA, Electroacupuncture; ELISA, Enzyme-Linked Immunosorbent Assay; ESR, Erythrocyte Sedimentation Rate; EX-LE/UE, Extra points of lower/upper extremities; FGF-2, Fibroblast Growth Factor-2; GB, Gallbladder meridian; GHQ-28, General Health Questionnaire-28; GIS, Global Improvement Score; HAQ, Health Assessment Questionnaire; IL, Interleukin; IGF-1, Insulin-like Growth Factor-1; KI, Kidney meridian; KOA, Knee Osteoarthritis; LKSS, Lysholm Knee Scoring Scale; LR/LV, Liver meridian; MA, Manual Acupuncture; MAPK, Mitogen-Activated Protein Kinase; MCH/MCHC/MCV, Mean Corpuscular Hemoglobin / Hemoglobin Concentration / Volume; MCP-1, Monocyte Chemotactic Protein-1; MDA, Malondialdehyde; MMP, Matrix Metalloproteinase; NF-κB, Nuclear Factor Kappa B; NE, Neutrophil; ODI, Oswestry Disability Index; PGE2, Prostaglandin E2; PLA2, Phospholipase A2; RA, Rheumatoid Arthritis; RF, Rheumatoid Factor; ROM, Range of Motion; SCV, Sensory Conduction Velocity; SOD, Superoxide Dismutase; SP, Spleen meridian; ST, Stomach meridian; sTNF-R1, Soluble Tumor Necrosis Factor Receptor-1; SUA, Serum Uric Acid; TCM, Traditional Chinese Medicine; TGF-β, Transforming Growth Factor-β; TNF-α, Tumor Necrosis Factor-α; VAS, Visual Analogue Scale; WOMAC, Western Ontario and McMaster Universities Osteoarthritis Index.
