# Supplementary material for: Immunomodulatory Effects of Acupuncture on Inflammatory Markers in Patients with Musculoskeletal Pain: A Systematic Review of Randomized Controlled Trials
Source: Muscles. 2026 May 8;5(2):36. doi: 10.3390/muscles5020036 (PMC13214626; doi:10.3390/muscles5020036)
Supplement: Supplementary file 1 [file muscles-05-00036-s001.zip › Supplementary Table S1.pdf]

Supplementary Table 1. Characteristics of included studies

S/N	Authors and Study Design	Country of study	Main diagnosis	Sample size	Age and gender ratio	Inclusion criteria	Exclusion criteria
1	David et al., 1999<br><br>Randomized, placebo-controlled cross-over trial	UK	RA	56 analysed (29 in Sequence A,<br><br>27 in Sequence B)	Age 18–75 yrs; median age:<br>61 (Seq A)<br>57 (Seq B);<br><br>gender not specified	Definite/classical RA per ACR criteria; on second-line therapy; stable drug regimen ≥3 months; aged 18–75 yrs; no intra-articular injections or pulse steroids in last 3 months	Previous acupuncture; anticoagulation; localized skin infection; use of other complementary therapy; fear of needles
2	Bernateck et al., 2008<br><br>RCT	Germany	RA	44 patients<br>Auricular EA + Autogenic Training: 19 completed<br><br>Autogenic Training: 19 completed	Auricular EA + AT: mean 56.3 ± 9.2 yrs; 3 M / 19 F<br><br>AT only: mean 57.4 ± 10.1 yrs; 4 M / 18 F	(1) Diagnosis of RA according to 1987 ACR criteria; (2) Functional class I–III; (3) Stable DMARD therapy; (4) Age 18–75 years	(1) Functional class IV; (2) Serious comorbid conditions (e.g., heart failure, renal failure); (3) Pregnancy/lactation; (4) Recent participation in another clinical trial
3	Jubb et al., 2008<br><br>RCT	UK	KOA	68 patients<br>(34 acupuncture 34 sham)<br><br>Completed: Acupuncture 23, sham 28	Acu: mean age 64.1 (29F)<br><br>Sham: mean age 66.1 (26F)	Symptomatic and radiographic KOA >6 months; inadequate response to ≥1 conventional treatments; not on surgical waitlist; aged >18	Previous acupuncture; pregnancy; other forms of arthritis; standard contraindications to acupuncture
4	Zanette et al., 2008<br><br>RCT	Brazil	RA	40 patients<br><br>Completed: 30 (AC: 16; Control: 14)	Age: AC 53.1 ± 12.4 years; Control 46.5 ± 9.9 years<br><br>Gender: AC 3 M/17 F; Control 0 M/20 F	ACR criteria for RA; Age 18–75; Active disease despite stable pharmacological therapy for ≥1 month; Able to complete HAQ and VAS	Prior acupuncture; sensory disturbances; infections; needle phobia; drug/alcohol abuse; pregnancy/lactation; anticoagulation or concurrent treatments; severe RA or advanced disease (class IV); serious comorbidities; conditions that preclude participation
5	Ahsin et al., 2009<br><br>RCT	Pakistan	Primary KOA	40 patients<br><br>(20 electro-acupuncture, 20 sham)	EA: mean age 51.05 ± 7.73 yrs; 7M/13F.	Age >40 yrs; primary OA knee per American College of Rheumatology criteria	Secondary OA; associated systemic arthropathies (e.g., RA, gout); on steroids or DMARDs; recent trauma at acupuncture area; pregnancy; intra-articular steroid

					Sham: mean age 51.45 ± 8.9 yrs; 5M/15F		injection in past 2 months; missed ≥2 EA sessions consecutively
6	Zukow et al., 2011<br><br>RCT	Poland	RA	70 patients<br><br>(35 acupuncture, 35 sham)	Treatment: 2M/33F, mean age 58.1 ± 11.5 yrs<br><br>Control: 3M/32F, mean age 57.6 ± 9.2 yrs	At least 4 out of 7 ACR criteria for RA, with criteria 1–4 present >6 weeks. No other therapies during the study period.	Not explicitly stated, but patients were instructed not to engage in other therapies during the study
7	Liang et al., 2012<br><br>RCT	China	RA	80 patients (Observation: 40; Control: 40). Completed: Observation 37, Control 40	Observation: 9M/31F, mean age 40 ± 9 yrs;<br><br>Control: 8M/32F, mean age 38 ± 9 yrs	Met 1987 ACR RA criteria; Age 17–70; Willing to discontinue other therapies and accept acupuncture + medication	RA in remission/inactive; deformed joints; multi-system damage or severe heart, liver, kidney, lung insufficiency; immunosuppressors in past 3 months; non-adherence to acupuncture leading to dropout
8	Lin et al., 2014<br><br>RCT	China	KOA	170 patients (264 knees)	Treatment group: 85 patients (39 unilateral, 46 bilateral)<br>41 M / 44 F<br>Age: 52.5 ± 8.6 to 60.4 ± 7.9 years<br><br>Control group: 85 patients (37 unilateral, 48 bilateral)<br>43 M / 42 F<br>Age: 51.4 ± 9.1 to 61.7 ± 8.9 years	Diagnosis of KOA per American College of Rheumatology criteria; Age >40 years; Pain/osteophytes + other clinical features; TCM diagnosis of 'stasis of the channels'	Age > 70 yrs; Rheumatoid arthritis, psoriasis, syphilitic neuropathy, ochronosis, metabolic osteopathy, acute trauma, other joint diseases; Pregnancy/lactation; Severe cardiovascular/hepatic/renal/mental disease; Recent treatments affecting outcomes
9	Ju et al., 2015<br><br>RCT	China	KOA	60 patients<br>(30 EA, 30 control)	EA: mean age 62.4 ± 7.3 yrs; 8M/22F.<br><br>Control: mean age 61.2 ± 6.9 yrs; 7M/23F	Diagnosis of KOA according to the American College of Rheumatology criteria; age 40–75 yrs	Other forms of arthritis; severe cardiovascular, hepatic, renal, or hematopoietic disorders; psychiatric illness; pregnancy or lactation; acupuncture within 1 month; corticosteroid use within 3 months
10	Arriaga-Pizano et al., 2020<br><br>RCT	Mexico	Low Back Pain (acute and chronic)	60 subjects:<br>24 athletes with CLBP (EA vs diclofenac)	All males<br>Age: 21 – 24 years (mean ≈ 22 years)<br><br>Groups:	Athletes with chronic LBP (6–12 months), related to overuse injuries, moderate intensity; normal labs	Female gender; smokers; major comorbidities; unclear pain origin; unable to comply with protocol

				36 healthy athletes (for EA vs sham or catecholamine analysis)	CLBP: EA vs diclofenac (12 + 12)<br>Acute LBP: EA vs sham EA (12 + 12)<br><br>Additional EA group for catecholamine measurement (n = 12)		
11	Deng et al., 2020<br><br>RCT	China	KOA	72 subjects	Stuck-needle:<br>14 M/22 F,<br>45.57 ± 5.82 yrs;<br><br>Regular acupuncture:<br>16 M/22 F<br>47.18 ± 3.42 yrs	(1) Met ACR (1995) diagnostic criteria for KOA; (2) No NSAIDs or immunosuppressants in past week; (3) No surgical treatment; (4) Voluntary consent and acceptance of regimen	(1) Severe joint deformities affecting treatment; (2) Local skin damage/infection; (3) Other arthritides (infectious, gout, RA, traumatic, TB); (4) Serious systemic diseases or organ dysfunction; (5) Extreme sensitivity or intolerance to needling
12	Shi et al., 2020<br><br>RCT	China	KOA (Kellgren–Lawrence grade II–III)	60<br>(EA n=28, MA n=30)	Mean age 58.9 ± 6.75 yrs; ~82% female	Age 45–75; knee pain ≥40 mm VAS for >6 months; radiographic KOA grade II–III (weight-bearing)	Severe comorbidities; inability to undergo trial procedures
13	Liu et al., 2022<br><br>RCT	China	Acute Gouty Arthritis (AGA)<br><br>TCM dampness-heat amassment pattern	90 randomized<br><br>(EA+DS: 30; Low-dose DS: 30; Conventional DS: 30); 87 completed	Male patients only<br>Age 35–70 years<br><br>EA+DS: median 58.0 yrs;<br>Low-dose DS: median 58.0 yrs;<br>Conventional DS: median 55.0 yrs	(1) Age 35–70 years; (2) Male; (3) Met 2015 ACR/EULAR GA diagnostic criteria; (4) Met TCM diagnostic criteria for AGA (dampness-heat amassment pattern); (5) Involvement of unilateral 1st MTP joint and/or foot/ankle joint; (6) Acute attack ≤24 hrs before admission; (7) VAS ≥4	(1) Allergy to NSAIDs; (2) Pacemaker, metal allergy, or severe needle phobia; (3) Acupuncture in prior 1 week; (4) AGA medication in prior 1 month; (5) Active GI disease or recent peptic ulcer (<30 days); (6) Severe cardiac, cerebrovascular, hepatic, renal, hematopoietic, or psychiatric disease
14	Liu & Wu, 2022<br><br>RCT	China	KOA	90 patients (30 per group)	EA group: 7M, 23F; Mean age: 58 ± 7 years<br>Acupuncture group: 8M, 22F; Mean age: 56 ± 6 years	(1) Diagnosed KOA per 2018 Guidelines; (2) Age 44–76; (3) No serious comorbidities; (4) Able to cooperate and consent	(1) Prior knee surgery or trauma; (2) Tuberculosis, RA, AS; (3) Pacemaker; (4) Pregnancy/lactation

					Medication group: 10M, 20F; Mean age: 62 ± 8 years		
15	Sun et al., 2022<br><br>RCT	China	KOA	81 patients (27 per group: Control, TCM, Combined)	Age 50–74 years (mean 61.31 ± 6.74), 47M/34F	(1) Met diagnostic criteria for KOA in Chinese guidelines; (2) Diagnosed by physical and imaging examination; (3) Signed informed consent; (4) No medication 1 month prior; (5) Conscious and able to cooperate	(1) Allergy; (2) Asthma; (3) Mental illness; (4) Severe heart failure; (5) Digestive ulcer; (6) Bone tumor or bone tuberculosis
16	Ye & Zou, 2022<br><br>RCT	China	Chondromalacia patellae	68 CP patients (34 per group)	Observation group: 13M, 21F; mean age 41.9 ± 8.1<br><br>Control group: 14M, 20F; mean age 42.4 ± 10.3	Diagnosis per Clinical Guidelines on Diagnosis and Treatment: Orthopedics<br>Aged 18–60 years<br>No medication/therapy in previous week<br>Unilateral CP	Meniscus/ligament injury<br>Bone tumor/tuberculosis<br>Bilateral CP<br>Pregnancy/lactation
17	Yang et al., 2023<br><br>RCT	China	KOA, Kellgren–Lawrence grade ≤ III	70 randomized; 67 completed (34 in intervention, 33 in control)	Intervention: 5M, 29F, mean age 51.6 ± 6.1<br>Control: 7M, 26F, mean age 51.7 ± 6.0	Age 40–75; met ACR criteria for KOA; met TCM diagnosis (QiPi); K-L grade ≤ III; no prior clinical trial participation	Prior knee surgery; meniscal/ligament/tuberculosis/infectious injury; coagulation issues or skin rashes; serious comorbidities (e.g., tumors, CVD); pregnancy or lactation
18	Cai et al., 2024<br><br>RCT	China	KOA	101 patients randomized; 96 completed (48 per group)	Acu-med group: 16M, 32F, mean age 60.3 ± 4.3;<br><br>Med-only group: 15M, 33F, mean age 60.8 ± 4.5	Diagnosis of KOA (per 2018 guidelines); wind-cold-dampness Bi-impediment (per TCM); age ≥50	Severe organ dysfunction; mental/cognitive disorder; knee fracture/tendon rupture; skin infection; allergy to trial meds; knee surgery; K-L grade IV
19	Wei et al., 2024<br><br>RCT	China	KOA (KL grade 2-3)	108 patients (48 males, 60 females)	48 males, 60 females; mean age 61.0±6.8 years balanced between groups	KOA per ACR criteria; knee pain ≥1 month; X-ray osteophytes; joint fluid test meeting OA standard; morning stiffness ≥30 min;	Other rheumatic diseases; prior knee surgery; mental disorders; coagulation or immune dysfunction; malignant tumors; serious cardiovascular or cerebrovascular disease

						bone fractures; unilateral KOA; KL grades 2-3	
20	Wu et al., 2025<br><br>RCT	China	Lumbar Disc Herniation (LDH)	100 patients (50 in each group)	Observation: 29M/21F, 55.4 ± 3.3 yrs<br>Control: 28M/22F, 55.2 ± 3.1 yrs	Diagnosis of LDH per clinical guidelines; aged 40–85; no relevant treatment in past 7 days	Spinal tumors, fractures, severe osteoporosis, infections, ankylosing spondylitis, immune/organ/coagulation dysfunction, psychiatric illness, pregnancy/lactation, prior lumbar surgery, poor compliance, or acupuncture contraindications

Abbreviation Legend:

ACR, American College of Rheumatology; AGA, Acute Gouty Arthritis; AS, Ankylosing Spondylitis; AT, Autogenic Training; CLBP, Chronic Low Back Pain; CP, Chondromalacia Patellae; CVD, Cardiovascular Disease; DMARD, Disease-Modifying Antirheumatic Drug; EA, Electroacupuncture; EULAR, European Alliance of Associations for Rheumatology; F, Female; M, Male; GA, Gouty Arthritis; K-L, Kellgren–Lawrence grading; KOA, Knee Osteoarthritis; LDH, Lumbar Disc Herniation; MA, Manual Acupuncture; MTP, Metatarsophalangeal Joint; NSAID, Non-Steroidal Anti-Inflammatory Drug; OA, Osteoarthritis; RA, Rheumatoid Arthritis; RCT, Randomized Controlled Trial; Seq A/B, Sequence A or B; SP, Spleen meridian; ST, Stomach meridian; GB, Gall Bladder meridian; LI, Large Intestine meridian; LV, Liver meridian; TCM, Traditional Chinese Medicine; VAS, Visual Analogue Scale.
